# „B-Probleme“ des nichttraumatologischen Schockraummanagements

**DOI:** 10.1007/s10049-022-00990-5

**Published:** 2022-03-10

**Authors:** Bernhard Kumle, Mark Michael, Andreas Wermke, Christoph Schmitz, Niels Hammer, Philipp Kümpers, Martin Pin, Michael Bernhard

**Affiliations:** 1 Klinik für Akut- und Notfallmedizin, Schwarzwald-Baar Klinikum, Klinikstr. 11, 78052 Villingen-Schwenningen, Deutschland; 2 Medical Life Science, Campus Schwenningen, Furtwangen University, Schwenningen, Deutschland; 3 Zentrale Notaufnahme, Universitätsklinikum Düsseldorf, Heinrich-Heine-Universität, Düsseldorf, Deutschland; 4 Interdisziplinäres Notfallzentrum, Kantonsspital Schaffhausen, Schaffhausen, Schweiz; 5 Institut für Klinische und Makroskopische Anatomie, Medizinische Universität Graz, Graz, Österreich; 6 Klinik für Orthopädie und Unfallchirurgie, Universität Leipzig, Leipzig, Deutschland; 7 Abteilung Medizintechnik, Fraunhofer-Institut für Werkstoff- und Umformtechnik, Dresden, Deutschland; 8 Medizinische Klinik D, Allgemeine Innere Medizin und Notaufnahme sowie Nieren- und Hochdruckkrankheiten und Rheumatologie, Universitätsklinikum Münster, Münster, Deutschland; 9 Zentrale Notaufnahme, Florence-Nightingale-Krankenhaus, Düsseldorf, Deutschland

**Keywords:** Atemwegsmanagement, Konservatives Schockraummanagement, Notfallbeatmung, Respiratorische Störung, Point-of-care-Ultraschalluntersuchung (POCUS), Airway management, Emergency ventilation, Respiratory insufficiency, Point-of-care ultrasonography (POCUS)

## Abstract

Beim Primary Survey des nichttraumatologischen Schockraummanagements kritisch kranker Patienten dient das ABCDE-Schema zur sofortigen Erkennung und Therapie vitaler Gefährdungen. „B-Probleme“ gehen mit einer Störung der Atmung einher und bedürfen einer sofortigen Behandlung. Die Pathogenese von „B-Problemen“ ist gerade im nichttraumatologischen Schockraum vielfältig. Klinische Untersuchung, Notfallsonographie und Kenntnisse in den Oxygenierungstechniken und der Beatmung stellen hier wichtige Bestandteile der Diagnostik und Therapie dar. Hierzu sind ein standardisiertes Vorgehen und regelmäßiges Training in der Notaufnahme von elementarer Bedeutung.

## Abstract (en)

In the primary survey of resuscitation room management in critically ill nontrauma patients, the ABCDE (airway, breathing, circulation, disability, exposure) approach is used for immediate recognition and treatment of life-threatening conditions. “B problems” are associated with respiratory failure and require immediate treatment. The pathogenesis is diverse, especially in the nontrauma resuscitation room. Clinical examination, emergency sonography and knowledge of oxygenation techniques and ventilation are important components of diagnosis and therapy. Standardized procedures and regular training in the emergency room are of fundamental importance.

## Epidemiologie

In einer Studie der Münchner Notaufnahme suchten 80 % der Patienten ohne Zuhilfenahme des Rettungsdienstes eine Notaufnahme auf [37]. Von diesen Patienten wird jeder siebte Patient auf eine Intermediate-care(IMC)- oder Intensivstation (ICU) aufgenommen. Pro 100.000 Einwohner werden damit rund 7 ICU/IMC-Betten/Tag benötigt. Das Verhältnis von kritisch kranken nichttraumatologischen Patienten zu Traumapatienten betrug 4:1 [37]. Diese Zahlen belegen die Relevanz der Schockraumversorgung für nichttraumatologisch kritisch kranke Patienten.

In den OBSERvE-Studien wurden erstmals prospektive, monozentrische Daten nichttraumatologisch kritisch kranker Schockraumpatienten analog zur Registererfassung von Traumapatienten erhoben [2, 11]. Diese Daten weisen bei nichttraumatologischen Schockraumzuweisungen in 26–29 % ein B‑Problem auf [11].

In einem akademischen Lehrkrankenhaus mit 756-Betten konnte gezeigt werden, dass rund 51 % der Patienten Diagnosen aufwiesen, die mit einer Dyspnoe einhergehen können (z. B. Pneumonie, COPD, kardiale Dekompensation, Lungenödem; [22]). Die pulsoxymetrische Sauerstoffsättigung bei Aufnahme war bei Verstorbenen im Vergleich zu überlebenden Patienten signifikant geringer (89 vs. 95 %, *p* = 0,0092; [22]). Das Leitsymptom „Dyspnoe“ wies in einer universitären Notaufnahme mit 18 % den höchsten Anteil an intensivpflichtigen Patienten und mit 9 % die höchste Mortalität auf [31]. Patienten mit einem B‑Problem sollte daher besondere Aufmerksamkeit gewidmet werden.

## Primary Survey – Schwerpunkt B-Problem

**Figure 1:**
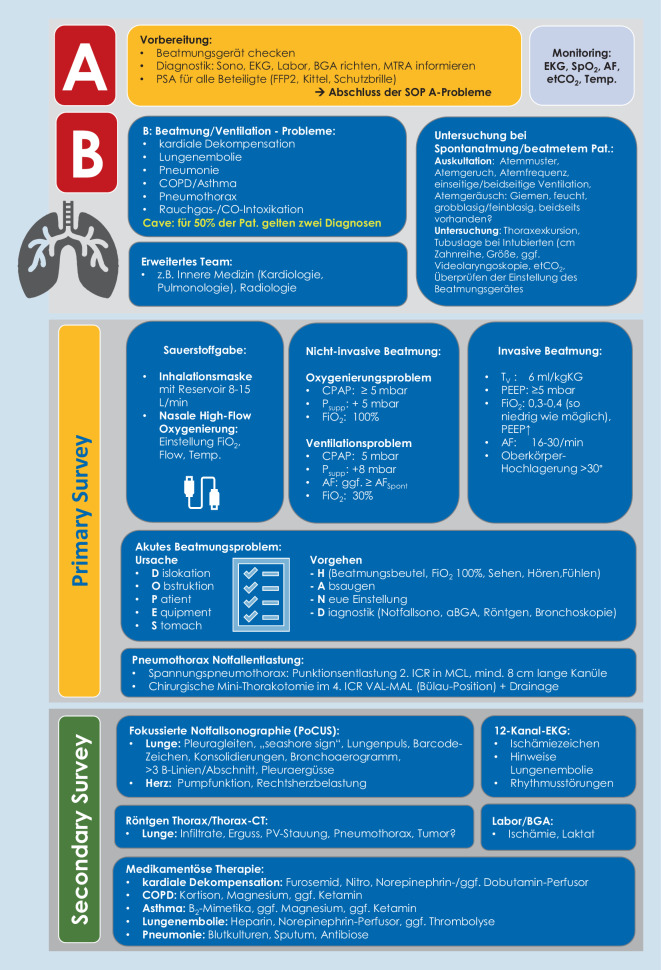

Nach der Beseitigung eines potenziellen A‑Problems [30] müssen B‑Probleme (Ventilations- und Gasaustauschstörungen) aufgrund der vielfältigen Pathologien als Ursache strukturiert anhand einer SOP abgearbeitet werden (Abb. 1).

### Klinische Untersuchung

Beim ersten Schritt wird eine suffiziente Atmung geprüft:

- **Thoraxinspektion:** einseitige Atembewegung oder Überblähung einer Thoraxhälfte (z. B. einseitiger Spannungspneumothorax)
- **Hautkolorit:** Hinweise auf Hypoxie (Zyanose) oder Intoxikation (z. B. CO-Vergiftung)
- **Atemmuster:** Bradypnoe, Tachypnoe und pathologische Atemmuster (z. B. „Kussmaul-Atmung“ [metabolische Azidose], „Cheyne-Stokes-Atmung“ [Schäden des Atemzentrums] oder „Schnappatmung“ [präfinal])
- Auskultation:
  - Beidseitige Auskultation der Lungen: fehlendes Atemgeräusch (Pneumothorax, Hämatothorax, einseitige Intubation) oder pathologische Atemgeräusche (Giemen, Brummen, Spastik, grob- oder feinblasige Rasselgeräusche)
  - Auskultation über dem Epigastrium direkt nach der endotrachealen Intubation beim ersten Beatmungshub, um eine ösophageale Fehlintubation zu erkennen
- **Weitere klinische Untersuchung:** Betrachtung von Hals, Thorax und Körperstamm → gestaute Halsvenen als Hinweis auf Perikarderguss oder Pneumothorax, Hautemphysem bzw. Krepitationen der Rippen und Hautknistern → Pneumothorax
- **(Invasive) Beatmung:** korrekte Maskengröße und Ausschluss einer Leckage bei nichtinvasiver Ventilation (NIV), korrekte Tubuslage, gutes Kapnographiesignal (Messung des exspiratorischen Kohlenstoffdioxids [CO_2_]).

### Monitoring

Die Überwachung der Vitalparameter ist ein wichtiger Bestandteil zur Erfassung eines B‑Problems. Gängige nichtinvasive Monitorfunktionen sind: Herzfrequenz, Blutdruck, Sauerstoffsättigung, Temperatur, exspiratorisches CO_2_ und Atemfrequenz. Die Atemfrequenz ist ein wichtiger Parameter bei der Beurteilung kritisch kranker Patienten und bietet einen Hinweise auf muskuläre Erschöpfung (z. B. bei COPD-Patienten), Pneumonie, metabolische Störungen oder das Vorliegen einer Sepsis.

Für die Erfassung der Sauerstoffsättigung stehen mehrere Arten von Sensoren zur Verfügung: Fingerclip, Ohr- oder Stirnsensoren. Die Anlage einer invasiven Blutdruckmessung gehört nicht zu den primären Maßnahmen der Versorgung, ist aber zur wiederholten arteriellen Blutgasanalyse (BGA) im Rahmen der Therapiekontrolle (Oxygenierung, Säure-Basen-Status, Elektrolyte, Laktat- und Hb-Kontrolle) oder zur engmaschigen Überwachung sinnvoll.

### Point-of-care-Ultraschalluntersuchung (POCUS)

Die Notfallsonographie/Point-of-care-Ultraschalluntersuchung ist ein wesentlicher Bestandteil der Diagnostik. Der Primary Survey dient ausschließlich dazu, ein vitales Problem zu erkennen (z. B. Spannungspneumothorax) und zu behandeln (z. B. Entlastungspunktion).

**Figure 2:**
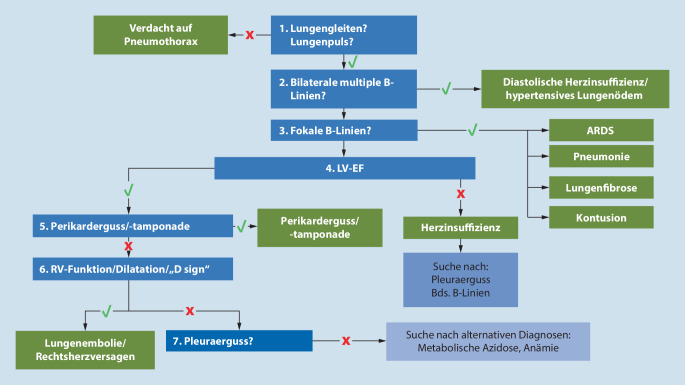

Bei einer akuten Atemnot/Dyspnoe können mittels sonographischer Notfalldiagnostik aus POC-Thoraxsonographie und POC-Echokardiographie konkrete Diagnosen und Differenzialdiagnosen identifiziert werden [15, 25, 34]:

- **POC-Thoraxsonographie**: Differenzierung von vorhandenem und fehlendem Lungengleiten (z. B. bei Pneumothorax; Abb. 2). Je nach Protokoll wird der Hemithorax in 3 bis zu 6 Quadranten eingeteilt (z. B. BLUE Protocol [25]). Weitere Schritte sind die Artefaktanalyse, welche Rückschlüsse auf Pathologien des Lungenparenchyms zulässt, sowie die Beurteilung der Thoraxwand (Tumoren, Hämatome) und der Pleuralinie (z. B. fragmentiert, verdickt; [15, 18, 24, 25]).
- **POC-Echokardiographie**: Erfahrene Untersucher können häufig mit verschiedenen Anlotungspunkten (subkostal, apikal, parasternal Längs- und Querachse) eine Ersteinschätzung der systolischen Funktion vornehmen (z. B. eingeschränkte linksventrikuläre Ejektionsfraktion, regionale Wandbewegungsstörungen, Rechtsherzbelastung, Perikarderguss oder relevante Klappenvitien).

### Blutgasanalyse (BGA)

Die arterielle BGA ist eine der schnellsten möglichen Laboranalysen, die auch schon innerhalb der ersten Minuten im Primary Survey zur Verfügung stehen [29]: pH-Wert, p_a_CO_2_ (Kohlendioxidpartialdruck im Blut), p_a_O_2_ (Sauerstoffpartialdruck im Blut), BE (Basenabweichung), HCO_3_^−^ (Bikarbonat) und S_a_O_2_ (Sauerstoffsättigung). pO_2_ (< 65 mm Hg) und S_p_O_2_ (< 90 %) geben einen Hinweis auf eine Hypoxämie, pCO_2_ (< 35 mm Hg, > 45 mm Hg), pH-Wert (< 7,35, > 7,35), BE (< −2 mmol/l, > +2 mmol/l) und Laktat (< 2 mmol/l) einen Hinweis auf eine Störung der Ventilation oder eine metabolische Störung [3]. Dies gilt vorwiegend für die arterielle sowie kapilläre BGA. In einer venösen Entnahme kann der pH, der pCO_2_ nur annäherungsweise und der pO_2_ gar nicht zur Beurteilung der Ventilation und Oxygenierung verwendet werden [29]. Daher sollten alle Patienten mit einem vermuteten „B-Problem“ immer eine arterielle oder kapilläre BGA erhalten.

### Sauerstofftherapie

Die Sauerstofftherapie gehört zu den primären Maßnahmen bei einem B‑Problem und erfolgt bei Spontanatmung über die Verabreichung von 1 bis 15 l O_2_/min und mit den Devices können unterschiedlich hohe inspiratorische Sauerstoffkonzentrationen (F_i_O_2_) erreicht werden:

- Nasenbrille mit 1–6 l O_2_/min: F_i_O_2_ 0,2–0,4
- Maske ohne Reservoir mit 7–15 l O_2_/min: F_i_O_2_ 0,4–0,6
- Maske mit Reservoir mit 7–15 l O_2_/min: F_i_O_2_ 0,7–0,9

Letztlich ist die tatsächliche inspiratorische Sauerstoffkonzentration abhängig von der Atemfrequenz, dem Atemzugvolumen und dem Inspirationsflow. Deshalb sind diese Werte nur als Annäherung zu verstehen.

### Nasale High-flow-Sauerstofftherapie

Bei der nasalen High-flow(NHF)-Sauerstofftherapie wird erwärmter (31–37 °C) und befeuchteter Sauerstoff mit Flussraten zwischen 30 und 70 l/min über eine nasale Doppelkanüle (Prongs) verabreicht. Die Sauerstoffkonzentration wird über einen Gasmischer differenziert abgegeben. NHF kann durch den hohen kontinuierlichen Fluss mehr Angebot liefern als der vom Patienten generierte inspiratorische Spitzenfluss (40–60 l/min). Die Erwärmung und Befeuchtung der Atemluft schafft eine gute Akzeptanz beim Patienten, da diese die Motilität der Zilien und die Viskosität des Schleims verbessern. Dies führt zu einer Abnahme des Atemwegswiderstands, einer geringen Bronchoreagibilität und Zunahme der Compliance durch verminderten Sekretstau sowie Verhinderung von Inkrustationen. Bei jeder Steigerung des „flow“ um 10 l/min wird der Atemwegsdruck bei offenem Mund durchschnittlich um zusätzlich ca. 0,6 cmH_2_O, bei geschlossenem Mund um 1,73 cmH_2_O erhöht [12]. Durch den positiven Atemwegsdruck wird die Oxygenierung weiter verbessert. Zudem kommt es durch den hohen Fluss zu einem „wash-out“ des nasopharyngealen Totraums mitsamt einer möglichen Verringerung des CO_2_.

### Nichtinvasive Beatmung

Unter nichtinvasiver Ventilation (NIV) versteht man die Anwendung eines kontinuierlichen positiven Atemwegsdrucks („continuous positive airway pressure“ [CPAP]) ohne/mit einer zusätzlichen Druckunterstützung (P_supp_) bei der Einatmung. Zusätzlich kann durch das Beatmungsgerät eine Atemfrequenz verabreicht werden, die nahe oder höher als die des Patienten ist, um die Atemmuskulatur zu entlasten. Die reine CPAP-Therapie wird meist bei reinen Oxygenierungsstörungen eingesetzt, die Druckunterstützung oder Vorgabe einer Atemfrequenz durch das Beatmungsgerät bei respiratorischer Erschöpfung der Atempumpe. Es gibt sehr einfache und differenzierte Schemata für die NIV-Anwendung [7, 23]. Die NIV kommt inzwischen bei fast allen Arten der respiratorischen Partial- oder Globalinsuffizienz zum Einsatz [40], aber auch als Bridging-Maßnahme oder Präoxygenierung vor der Atemwegssicherung [39].

### Invasive Beatmung

Die endotracheale Intubation und invasive Beatmung ist bei kritisch kranken, nichttraumatologischen Patienten der Goldstandard bei schweren respiratorischen Störungen. Als Komplikationen können pulmonale Schädigungen auftreten, die entweder mit der Beatmung assoziiert („ventilator-associated lung injury“ [VALI]) oder durch die Beatmung hervorgerufen („ventilator-induced lung injury“ [VILI]) sind. Bereits im Schockraum sollen daher Beatmungsstrategien angewendet werden, die keine weiteren Schäden hervorrufen.

Die Anwendung eines strikten lungenprotektiven Beatmungsregimes kann bereits in der Notaufnahme die Krankenhausmortalität senken ([10]; Abb. 1).

Bei Auftreten von Komplikationen im Rahmen der invasiven Beatmung ist ein strukturiertes Vorgehen zur Fehlerbehebung sinnvoll (Akronym „DOPES“ und „HAND“; Tab. 1).

**Table 1:** 

*Ursache*		
**D**	Dislokation	Tubuslage korrekt? CO_2_? Tubus an Respirator angeschlossen? Leck im Bereich des Schlauchsystems? Ösophageale Fehllage? Der Tubus kann auch nach korrekter Intubation bei unzureichender Sicherung in den Ösophagus abrutschen!
**O**	Obstruktion	Tubus abgeknickt? Sekret verlegt? Fixierung? Patient beißt auf den Tubus? Inspektion des Tubus, bei Bedarf Absaugung des Tubus
**P**	Pneumothorax	Umgehende Auskultation (einseitiges Atemgeräusch?); wenn möglich Sonographie („barcode sign“ vorhanden?); bei Pneumothorax Entlastungspunktion/Thoraxdrainage
	„Pressure“	Plötzlich auftretende Zunahme des Beatmungsdrucks? → einseitige Tubusfehllage → immer unter Sicht korrigieren!
		Punktuell, als Spitze auftretender Anstieg des Beatmungsdrucks? → Hinweis auf „Pressen“ des Patienten gegen die Beatmung → Narkosevertiefung
**E**	„Equipment“	Bei Beatmungsproblem sofort Beatmungseinstellungen überprüfen! Im Zweifel manuelle Beatmung mit separatem Beatmungsbeutel! Technische Probleme detektieren, z. B. Dislokation von Schläuchen, eingeklemmte Leitungen, Fehleinstellungen am Beatmungsgerät usw.
**S**	„Stomach“	Cave: Überblähung des Magens bei Larynxtubus – Kontrolle des Cuffs! → Auskultation, ggf. Neuintubation! Hinweise: abrupter CO_2_-Abfall, steigender Beatmungsdruck Auch bei Säuglingen und Kleinkindern Überblähung, insbesondere nach prolongierter Maskenbeatmung → Anlage einer Magensonde
*Vorgehen*		
**H**	Hand	Beatmungsbeutel, 100 % O_2_ verabreichen, Sehen/Hören/Fühlen
**A**	Absaugen	endotracheal, Magen, ggf. Magensonde bei Überblähung
**N**	Neue Einstellung	Beatmungsgerät
**D**	Diagnostik	Notfallsono, BGA, Röntgen, Bronchoskopie

### Zustandsverschlechterung bei intubierten Patienten

Bei Schockraumaufnahme wird unmittelbar der Atemweg überprüft. Noch vor Übernahme des Patienten erfolgt die Registrierung der Sauerstoffsättigung und des kapnographisch ermittelten etCO_2_-Werts im Monitoring des Rettungsdiensts („5 second round“). Nach der Umlagerung wird der Patient zunächst mit dem Beatmungsbeutel ventiliert, um die Mechanik der Ventilation zu prüfen und die Atemnebengeräusche besser auskultieren zu können. Ein seitendifferentes Atemnebengeräusch kann auf eine Störung der Atemfunktion hindeuten (z. B. Pneumothorax oder Hämatothorax), die mittels Notfallsonographie verifiziert wird.

Bei der Verwendung eines supraglottischen Atemwegs ist grundsätzlich der geeignete Moment des Wechsels auf eine endotracheale Intubation (sog. Exchange-Manöver) kritisch zu prüfen. Bei Problemen (z. B. S_p_O_2_-Abfall, CO_2_-Anstieg/-Abfall, Beatmungsdruckanstieg oder -abfall, Alarmierung des Beatmungsgeräts) ist ein sofortiger Wechsel indiziert. Bei suffizient einliegendem supraglottischem Atemwegshilfsmittel kann der Wechsel auf einen späteren Moment im Rahmen der Schockraumversorgung verschoben werden. Die Autoren plädieren dafür, bei einliegendem supraglottischem Atemweg immer vor Verlassen des Schockraums eine definitive Atemwegssicherung mittels endotrachealer Intubation durchzuführen, hier sollte eine standardisierte Technik (z. B. mittels Videolaryngoskopie) angewandt werden, um die Sicherheit zu erhöhen [19].

## Häufige Erkrankungen mit „B-Problem“

**Figure 3:**
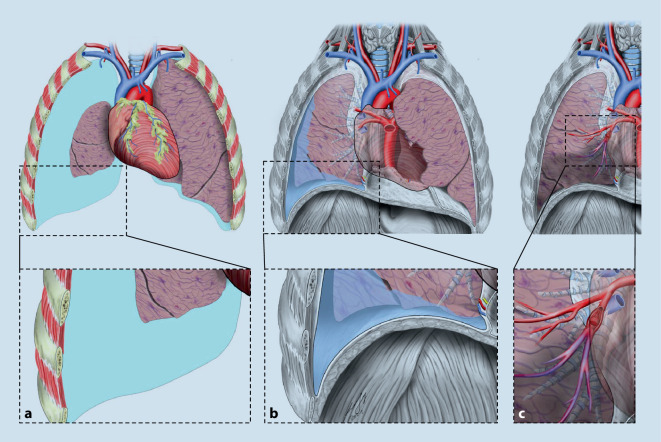

Zahlreiche kritische Erkrankungen (Abb. 3 und 4) können einem „B-Problem“ im nichttraumatologischen Schockraummanagement zugrunde liegen. Die häufigsten Erkrankungen sollen in der nachfolgenden Übersicht dargestellt werden.

**Figure 4:**
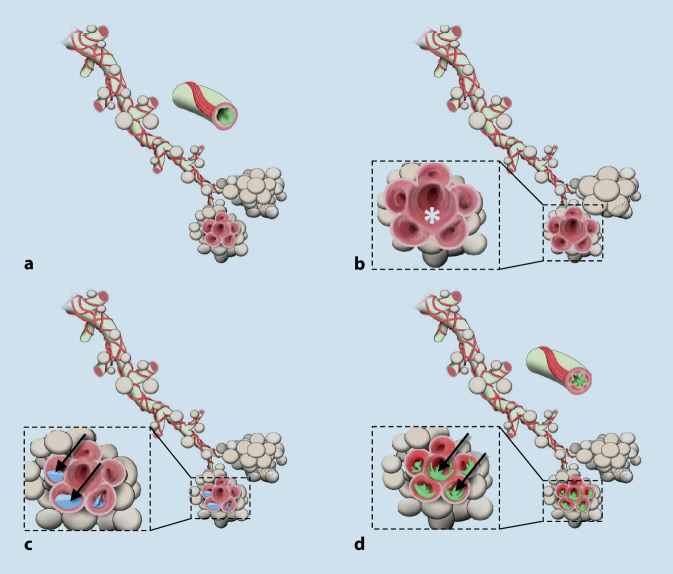

**Figure 5:**
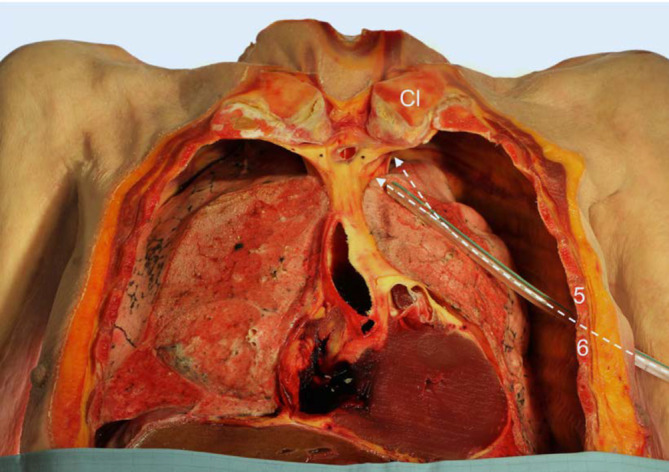

### Asthma

Akute Exazerbationen bronchoobstruktiver Krankheiten sind eine häufige Ursache für ein akutes „B-Problem“. Leitsymptome sind die schwere Atemnot, deutlich verlängertes Exspirium, Tachypnoe und Einsatz der Atemhilfsmuskulatur. Klinische Zeichen sind: pulmonale Überblähung, Husten, zähes, schwer zu mobilisierendes Bronchialsekret, Pulsus paradoxus, Auskultation: Giemen, Brummen, Pfeifen (Bronchospasmus). Eine lebensbedrohliche Situation entwickelt sich aufgrund respiratorischer Erschöpfung mit Hypoventilation und konsekutiver Hyperkapnie, auskultatorisch findet sich das Bild einer „silent lung“.

Ziele der Behandlung sind eine Verbesserung der Ventilation und des pulmonalen Gasaustauschs (Ziel‑S_p_O_2_: 92–95 %).

Grundpfeiler der medikamentösen Therapie der bronchialen Obstruktion ist die Gabe von Bronchodilatatoren (Beta-2-Mimetika und Anticholinergika) und Steroiden. Inhalative Beta-2-Mimetika haben einen schnellen Wirkeintritt und ein geringes Nebenwirkungsprofil, eine alleinige Therapie mit Anticholinergika ist nicht effektiv. Die Gabe von 1 bis 2 mg/kgKG i.v. Prednisolonäquivalent gehört mit zur Initialtherapie des akuten Asthmaanfalls [5]. Bronchodilatatorische Effekte hat auch die intravenöse Gabe von Magnesium (2 g/20 min i.v.). Theophyllin hat eine geringe therapeutische Breite, bringt keinen zusätzlichen Effekt zu inhalativen Beta-2-Mimetika (Nebenwirkung: schwere Rhythmusstörungen; [5]), und man sollte deshalb auf die Theophyllingabe verzichten. Definitiv ist eine Kurzinfusion der Bolusinjektion vorzuziehen (initial 5 mg/kgKG als Kurzinfusion). Beta-2-Mimetika können auch parenteral verabreicht werden (z. B. Terbutalin 0,25–0,5 mg s.c. alle 4 h oder Reproterol 0,09 mg langsam intravenös [Wiederholung nach 10 min möglich]). Aufgrund des Risikos auf eine Verminderung der Ventilation gilt die Sedierung als kontraindiziert [5]. Zur Anxiolyse können im Einzelfall auch sedierende Medikamente wie z. B. 1–2 mg Midazolam i.v. oder 2,5–5 mg Morphin sinnvoll sein.

### Akut exazerbierte chronisch-obstruktive Lungenerkrankung

Im Gegensatz zum Asthma ist die chronisch-obstruktive Lungenerkrankung (COPD) ein langsam progredientes Krankheitsbild mit einer nicht reversiblen Verengung der Atemwege und einer Destruktion des Lungenparenchyms (Lungenemphysem). Eine akute Exazerbation (AECOPD, Abb. 4b) mit akuter Einschränkung der Lungenfunktion ist durch eine Zunahme der bronchialen Entzündung, häufig auf dem Boden einer viralen oder bakteriellen Infektion, gekennzeichnet.

Das Leitsymptom ist eine meist über Tage progrediente, schwere Atemnot mit Husten und ggf. mit zunehmend purulentem Sputum. Klinisch zeigen sich der Einsatz der Atemhilfsmuskulatur, Lippenbremse und Akrenzyanose. Aufgrund der chronischen Überblähung ist das Atemzugvolumen des Patienten deutlich verringert. Kompensatorisch entwickelt der Patient eine Tachypnoe. Auskultatorisch findet sich die sogenannte „silent lung“. Die zunehmende Instabilität des COPD-Patienten bestehend aus Tachypnoe, Tachykardie, Unruhe und eine zunehmende Vigilanzstörung (Hyperkapnie) deuten auf eine dekompensierende respiratorische Situation hin. Die Therapie entspricht weitestgehend der des Asthmaanfalls, deshalb wird im Folgenden nur auf die Besonderheiten der COPD eingegangen.

Die Basistherapie besteht aus der Gabe von Sauerstoff (Ziel: S_p_O_2_: 90 %). Eine arterielle BGA erkennt Hyperkapnie und Azidose im Rahmen einer Dekompensation.

Bei der akuten Exazerbation der COPD ist die inhalative Beta-2-Mimetika-Gabe das Mittel der ersten Wahl [38]. Die intravenöse Gabe von Beta-2-Mimetika ist beim COPD-Patienten mit häufig kardialen Nebenerkrankungen kritischer zu sehen und möglicherweise bedrohlicher.

Die Gabe von 50 bis 100 mg Prednisolonäquivalent ist bei der akuten Exazerbation der COPD uneingeschränkt empfehlenswert.

Der CPAP mit zusätzlichem P_supp_ hat einen hohen Stellenwert bei der schweren AECOPD [40]. Eine primäre Intubation des COPD-Patienten sollte vermieden werden, da sie prognostisch ungünstig und mit einer erhöhten Mortalität vergesellschaftet ist [38]. Abbruchkriterien für eine NIV und ggf. Durchführung einer Intubation sind, wenn nach 1–2 h folgende Parameter erfüllt sind: Zunahme der Dyspnoe, Verschlechterung der Vigilanz, Zunahme des P_a_CO_2_, Zunahme der Atemfrequenz, Abnahme des pH, Abnahme von S_a_O_2_ ≤ 85 %, Zunahme der Herzfrequenz [6].

### Pneumonie

Die schwere bilaterale Pneumonie (Abb. 4d) ist gekennzeichnet durch eine schwere Atemnot, Tachypnoe, Hypoxie, Vigilanzstörung und eventuell eine Kreislaufinstabilität. Dazu kommen die klassischen Symptome wie Fieber, Krankheitsgefühl, Schüttelfrost, Hypothermie, Exsikkose, Husten ± Auswurf, atemabhängiger Thoraxschmerz (Pleuritis) sowie Begleiterguss, auskultierbare pulmonale Rasselgeräusche oder eine bereits mit bloßem Ohr hörbar rasselnde Atmung. Mit der Lungensonographie können einseitige oder beidseits asymmetrische Pleuraergüsse oder direkt ein Infiltrat gesehen werden [8, 27, 28, 32]. Bei sonographischen oder radiologischen Hinweisen auf einschmelzende Infiltrate, Abszesse oder ein Pleuraempyem sollte immer eine Computertomographie des Thorax erfolgen [8].

Bei Hypotonie werden balancierte Elektrolytlösungen und Vasopressoren intravenös eingesetzt (1. Wahl Noradrenalin, MAP-Zielwert: 65 mm Hg, Laktatnormalisierung; [4, 8]).

Zur mikrobiologischen Diagnostik gehören immer mindestens 2 Paar Blutkulturen, dazu ergänzend Atemwegsabstriche auf saisonale/aktuelle Erreger (Influenza, COVID-19), Sputum oder Trachealsekret mit Gram-Färbung, Pneumokokken- und Legionellen-Ag im Urin sowie je nach Anamnese (Tierkontakt, Reisen)/Epidemiologie/Klinik (atypische Infiltrate, Immunsuppression) eine erweiterte Diagnostik (z. B. Chlamydien‑/Mykoplasmenantikörper im Serum, Pilzkulturen, bronchoalveoläre Lavage; [8]).

Im Schockraum sollte direkt (< 1 h) nach Abnahme der Blutkulturen eine intravenöse antimikrobielle Therapie (i.v.-Kombinationstherapie aus Breitspektrum-Betalaktam [z. B. Tazobactam/Piperacillin] und einem Makrolid) begonnen werden [4].

Die Wahl der Medikation richtet sich nach der Schwere des Krankheitsbilds, individuellen Risikofaktoren (z. B. Immunsuppression, Vorkommen multiresistenter Erreger [z. B. multiresistente *P. aeruginosa*]) und der aktuellen lokalen Epidemiologie (z. B. Influenza/COVID-19/Legionellenausbruch; [8]).

Des Weiteren kann bei Diagnose „Pneumonie“ ein begleitender großer Pleuraerguss diagnostisch punktiert und bei Nachweis eines komplizierten Ergusses (klar, septiert, Bakteriennachweis oder pH zwischen 7,0 und 7,2) oder eines Pleuraempyems (trüb, pH < 7,0) mittels Drainage entlastet werden. Beim Pleuraempyem wird neben der Drainage ein Breitspektrum-Betalaktam (alternativ Moxifloxacin) empfohlen.

Die Prognose und das Risiko der Pneumonie können mit dem CRB-65-Score abgeschätzt werden [8]. Major-Kriterien für ein hohes Risiko sind eine notwendige invasive Beatmung oder ein Katecholaminbedarf. Minor-Kriterien sind schwere Hypoxie mit P_a_O_2_ ≤ 55 mm Hg unter Raumluft, Atemfrequenz ≥ 30/min, multilobuläre Infiltrate im Röntgen, neue Bewusstseinstrübung, Hypotension mit notwendiger aggressiver Volumentherapie, akutes Nierenversagen mit Harnstoff ≥ 200 mg/l, Leukopenie (< 4000/µl), Thrombopenie (< 100.000/µl) und Hypothermie (< 36 °C). Ein Major-Kriterium oder ≥ 2 Minor-Kriterien und ein erhöhtes Letalitätsrisiko sprechen für eine weitere intensivmedizinische Betreuung. Ein unabhängiger Risikoprädiktor ist eine Laktaterhöhung [8].

### Lungenembolie

Die Lungenarterienembolie (LAE, Abb. 3c) weist häufig als Leitsymptome Dyspnoe, Thoraxschmerz, Synkope, Hämoptysen, (obstruktiver) Schock oder gar Herz-Kreislauf-Stillstand auf [20].

Gerade bei hämodynamisch instabilen Patienten mit der Differenzialdiagnose LAE sollten immer eine fokussierte Echokardiographie zur Detektion von Rechtsherzbelastungszeichen (z. B. erweiterter rechter Ventrikel mit RV/LV-Durchmesser > 1, reduzierte rechtsventrikuläre Funktion [TAPSE < 16 mm], „D sign“, „early systolic notching“) sowie eine beidseitige Kompressionssonographie der V. femoralis und V. poplitea durchgeführt werden.

Auch kardiale Biomarker (z. B. Troponin, NT-proBNP) können auf eine Rechtsherzbelastung hinweisen. Die D‑Dimere sind bei einer LAE in der Regel erhöht, Normwerte schließen eine zentrale Lungenarterienembolie bei geringer/mittlerer Prätestwahrscheinlichkeit (Wells-Score) nahezu aus. Elektrokardiographische Zeichen einer LAE sind: Sinustachykardie, T‑Negativierung V1–V4 und II/III/aVF, Rechtsschenkelblock, P pulmonale, S1/Q3-Typ oder auch Vorhofflimmern. Diese Zeichen sind aber weder sensitiv noch spezifisch genug. Mittel der Wahl zum definitiven Nachweis einer LAE ist daher die CT-Angiographie der Pulmonalarterien.

Bereits bei hinreichendem klinischem Verdacht (Wells-Score > 6) auf eine akute LAE, spätestens jedoch bei definitiver Diagnose sollte eine systemische Antikoagulation initiiert werden (bei hämodynamisch stabilen Patienten: niedermolekulare Heparine [LMWH] oder neue orale Antikoagulanzien [NOAK], bei instabilen Patienten präferenziell unfraktioniertes Heparin [UFH], damit bei einer Verschlechterung eine systemische Lysetherapie erfolgen kann). Hierzu wird Alteplase (rtPA) 100 mg über 2 h gegeben. Bei extremer hämodynamischer Instabilität oder unter CPR können auch 0,6 mg/kg über 15 min verabreicht werden.

Eine LAE wird durch die Hypoxämie und die Rechtsherzbelastung vital bedrohlich. Beides kann zu einem Herz-Kreislauf-Stillstand führen. Die Hypoxämie wird symptomatisch durch eine O_2_-Gabe (Nasenbrille/-maske, NHF, NIV bis invasive Beatmung) behandelt. Eine Narkose sollte wegen der drohenden hämodynamischen Instabilität nur im Notfall und durch qualifiziertes Fachpersonal erfolgen. PEEP und Spitzendruck sollten möglichst klein gewählt werden (Tidalvolumen ~6 ml/kg Idealgewicht und endinspiratorischer Plateaudruck < 30 cmH_2_O).

Zur Stabilisierung des Kreislaufs bei Rechtsherzversagen werden Noradrenalin und Dobutamin und bei einer Reanimation wird Adrenalin appliziert. Falls verfügbar und indiziert, kann eine venoarterielle ECMO zur Überbrückung bis zur Rekanalisation angeschlossen werden.

Sollte eine Kontraindikation für eine systemische Lysetherapie bestehen und die entsprechende Expertise vorhanden sein, ist eine chirurgische Embolektomie empfohlen oder alternativ eine perkutane Katheterintervention zu bedenken.

### Akute Herzinsuffizienz/Lungenödem

Die Mortalität der Herzinsuffizienz (Abb. 3b,c) beträgt > 50 %. In den bisherigen Daten zu nichttraumatologischen Schockraumkollektiven fanden sich die Diagnosen Herzinsuffizienz und Lungenödem in 16 % bzw. 13 % der Fälle [2, 22].

Nur 5–8 % der Patienten mit akuter Herzinsuffizienz erfüllen formal die Kriterien eines kardiogenen Schocks. Hinweise auf eine kardiale Dekompensation gibt es bereits bei der körperlichen Untersuchung: meist grobblasige Rasselgeräusche beidseits bei der Auskultation, gestaute Halsvenen, Beinödeme oder Anasarka. Kaltschweißige, graue Haut, Rekapillarisierungszeit > 3 s und Blutdruck < 90 mm Hg sind Hinweise auf einen bereits bestehenden kardiogenen Schock.

Neben der klinischen Untersuchung ist die zielführendste Diagnostik die fokussierte Thoraxsonographie und Echokardiographie (z. B. eine verminderte Pumpfunktion, Kinetikstörungen, schwerwiegende Klappendysfunktionen oder eine dilatierte V. cava ohne Atemmodulation; Abb. 2). Ergänzend sind ein Röntgen des Thorax und NT-proBNP hilfreich, ein normwertiges NT-proBNP spricht gegen eine kardiale Dekompensation.

Die Akuttherapie besteht bei schwerer Oxygenierungsstörung primär in der extrem niederschwelligen CPAP-Therapie [40]. Eine inspiratorische Druckunterstützung (P_supp_) ist selten notwendig. Bei milderen Oxygenierungsstörungen ist die Gabe von Sauerstoff, in der Regel über eine Reservoirmaske, ausreichend. Die medikamentöse Therapie der akuten Herzinsuffizienz ist abhängig davon, ob eine Hypotension oder Hypertension vorliegt. Bei Patienten mit einer Hypotension bzw. im kardiogenen Schock ist die Gabe von Dobutamin indiziert.

Die hypertensive akute Herzinsuffizienz mit akutem Lungenödem wird präferenziell mit Vasodilatatoren zur Vor- und Nachlastsenkung (z. B. Nitroglycerin, Isosorbiddinitrat) behandelt. Schleifendiuretika (z. B. Furosemid) kommen bei Überwässerung zum Einsatz. Die Indikation für eine Pleurapunktion besteht bei respiratorischer Insuffizienz und einem großen Pleuraerguss (> 300 ml; [17]). Liegt ein persistierendes Hypoperfusionssyndrom bzw. persistierender Schock mit „low cardiac output“ vor, reicht die medikamentöse Therapie oft nicht mehr aus. Hier kommen in speziellen Zentren extrakorporale Unterstützungsverfahren zur Anwendung. Die jahrzehntelang verwendete intraaortale Gegenpulsation (IABP) wird seit der IABP-Schock-II-Studie [36] kaum mehr eingesetzt. Abgelöst wurde sie durch den Einsatz von perkutan implantierbaren Axialpumpen (z. B. Impella RP®, Abiomed) oder Extracorporeal-cardiac-life-support-Systemen (ECLS).

### Pneumothorax

Ein Pneumothorax oder gar ein Spannungspneumothorax ist nicht immer nur die Folge eines Traumas, sondern kann entweder spontan (häufiger bei jungen Patienten) oder auch im Rahmen einer Überblähung einzelner Lungenareale (z. B. COPD) auftreten (Abb. 3a).

Beim Spannungspneumothorax ist die schnellste Art der Entlastung die notfallmäßige Entlastungspunktion in Monaldi-Position (2. Interkostalraum in der Medioklavikularlinie) oder in Bülau-Position (4./5. Interkostalraum, vordere/mittlere Axillarlinie) (Abb. 5) mit einer speziellen und ausreichend langen 14 G‑Dekompressionsnadel, immer gefolgt von einer Minithorakotomie mit Anlage einer Thoraxdrainage (≤ 14 Charrière) unter sterilen Kautelen [35].

### Metabolische Azidose

Als wichtige Differenzialdiagnose der Dyspnoe führt die metabolische Azidose (BGA: Kombination aus niedrigem CO_2_, pH, „base excess“ und Bikarbonat, [21]) zu einer Hyperventilation mit subjektiver Dyspnoe. In schweren Fällen kann die anhaltend hohe Atemfrequenz, zusammen mit einer kognitiven Beeinträchtigung durch den zugrunde liegenden Pathomechanismus (Hypotonie bei Schock, Intoxikation), zum respiratorischen Versagen führen. Die hohe Atemfrequenz ist der physiologische Kompensationsversuch durch Abatmung von CO_2_.

Die Ursachen für eine metabolische Azidose (Merkspruch KUSSMAUL: **K**etoazidose, **U**rämie, **S**alicylate, **M**ethanol, Ethylenglycol, **L**aktat) sind vielfältig [26].

Die Therapie richtet sich hier vor allem nach der Ursache der Azidose: bei diabetischer Ketoazidose: Volumengabe und intravenöse Insulingabe [1], bei Organischämie: Verbesserung der Gewebeoxygenierung [33], bei Intoxikationen: Antidotgabe oder auch Hämodialyse [14]. Die Gabe von Natriumbikarbonat kann vorübergehend eine Normalisierung des pH bewirken. Es gibt wenig Evidenz für einen therapeutischen „benefit“, mit Ausnahme der urämischen Azidose bei akuter Niereninsuffizienz [16, 26] oder bei Verlust oder Verbrauch von Bikarbonat.

### Rauchgasintoxikation

Hauptproblematik bei inhalativen Brandunfällen sind lokale thermische und toxische Schädigungen an Tracheal- und Bronchialsystem (lebensbedrohliche Oropharynxödeme, Bronchialwandödeme oder Schäden an den Alveolarwänden) bei z. B. Verpuffungen und die verminderte Sauerstoffutilisation durch Bindung von Kohlenmonoxid (z. B. Auspuffgase, Öfen, defekte Gasthermen, Wasserpfeifen [Shisha]) an Hämoglobin.

Bei Rauchgasintoxikationen stellt das Inhalationstrauma den Hauptfaktor für eine erhöhte Mortalität dar. Die Bandbreite der Symptome einer akuten Rauchgasintoxikation reicht in Abhängigkeit von Expositionszeit und Konzentration des Gases von leichten Symptomen (z. B. Kopfschmerzen, Schwindel) über pektanginöse Beschwerden mit Erhöhung der kardialen Biomarker insbesondere bei kardial vorerkrankten Patienten bis hin zum Tod. Die toxische Schädigung, die durch Reizgase an den unteren Atemwegen verursacht wird, kann mit einer bis zu 36-stündlichen Verzögerung auftreten.

Neben Anamnese, Auskultation, Inspektion des Mund- und Rachenraums und der Suche nach Verbrennungsspuren im Gesicht (z. B. versengte Augenbrauen) kann die Bestimmung des Carboxyhämoglobinwerts (CO-Hb) in einer BGA Auskunft über das Ausmaß der Schwere der CO-Vergiftung geben.

Die Schwere der CO-Intoxikation ist nicht allein mithilfe des in der BGA ermittelten CO-Hb-Werts zu diagnostizieren, sondern nur im Kontext mit Anamnese und klinischem Erscheinungsbild.

Das Hauptziel der Therapie bei CO-Intoxikationen ist die Elimination von CO, hierzu wird so früh wie möglich hoch dosiert Sauerstoff verabreicht. Die Elimination von CO ist umso effektiver, je höher der angebotene Sauerstoffpartialdruck ist. In der Präklinik und bei Ankunft im Schockraum bedeutet dies bei wachen Patienten die bevorzugte Anwendung einer NIV-Therapie mit 100 % F_i_O_2_ [9]. Eine hyperbare Sauerstofftherapie (HBO) kann die Eliminationshalbwertszeit weiter signifikant verkürzen. „*Bei den Anzeichen einer schweren Kohlenmonoxidvergiftung (u.* *a. fortgesetzte Bewusstseinsstörungen, metabolische Azidose, respiratorische Insuffizienz und/oder kardiale Ischämie) sowie bei Schwangerschaft sollte im Erwachsenenalter (18 Jahre) eine hyperbare Sauerstofftherapie innerhalb der ersten 6* *h durchgeführt werden*“ [9].

In Abhängigkeit des Schädigungsmusters durch die Inhalation besteht bei schweren thermischen Schäden der oberen Atemwege mit inspiratorischem Stridor und Heiserkeit die Indikation zur frühzeitigen Intubation, da mit einer Progression des Ödems zu rechnen ist. Bei leichteren Symptomen kann die Inhalation mit Adrenalin erfolgen. Bei Hinweisen auf eine chemisch-toxische Schädigung der unteren Atemwege sollte eine Inhalation mit Beta-2-Mimetika erfolgen.

## Fazit für die Praxis

- „B-Probleme“ im nichttraumatologischen Schockraummanagement stellen mit 26–28 % eine der führenden Problematiken dar.
- Das Leitsymptom Dyspnoe und respiratorische Störungen sind mit einer hohen innerklinischen Letalität assoziiert.
- Aufgrund der vielfältigen Ursachen der „B-Probleme“ sind fundierte Kenntnisse in der Pathophysiologie der Erkrankungen, der Diagnostik und Therapie obligat.
- Die Notfallsonographie ist mit der POC-Thoraxsonographie und POC-Echokardiographie die Schlüsseldiagnostik zur Differenzierung der Diagnosen und Einleitung der spezifischen Therapien.
- Durch den häufigen Einsatz der nichtinvasiven und invasiven Beatmung sind auch Schulungen im Umgang mit diesen Techniken, Indikationsstellung und Grenzen der Verfahren notwendig.
